# New Paralogs of the *Heliothis virescens ABCC2* Transporter as Potential Receptors for Bt Cry1A Proteins

**DOI:** 10.3390/biom14040397

**Published:** 2024-03-26

**Authors:** Daniel Pinos, Anabel Millán-Leiva, Juan Ferré, Patricia Hernández-Martínez

**Affiliations:** 1 Departamento de Genética, Instituto de Biotecnología y Biomedicina (BIOTECMED), Universitat de València, 46100 Burjassot, Spain; daniel.pinos@uv.es (D.P.); anabel.millan@uv.es (A.M.-L.); juan.ferre@uv.es (J.F.); 2 Centro de Protección Vegetal y Biotecnología, Instituto Valenciano de Investigaciones Agrarias (IVIA), Moncada, 46113 Valencia, Spain

**Keywords:** *Bacillus thuringiensis*, insecticidal proteins, receptors, tobacco budworm, docking analysis

## Abstract

The ATP-binding cassette (ABC) transporters are a superfamily of membrane proteins. These active transporters are involved in the export of different substances such as xenobiotics. ABC transporters from subfamily C (ABCC) have also been described as functional receptors for different insecticidal proteins from *Bacillus thuringiensis* (Bt) in several lepidopteran species. Numerous studies have characterized the relationship between the ABCC2 transporter and Bt Cry1 proteins. Although other ABCC transporters sharing structural and functional similarities have been described, little is known of their role in the mode of action of Bt proteins. For *Heliothis virescens*, only the ABCC2 transporter and its interaction with Cry1A proteins have been studied to date. Here, we have searched for paralogs to the *ABCC2* gene in *H. virescens*, and identified two new ABC transporter genes: *HvABCC3* and *HvABCC4*. Furthermore, we have characterized their gene expression in the midgut and their protein topology, and compared them with that of ABCC2. Finally, we discuss their possible interaction with Bt proteins by performing protein docking analysis.

## 1. Introduction

ATP-binding cassette (ABC) transporters are a superfamily of transmembrane proteins that can be found in all kind of organisms. These active transporters are involved in the export of allocrites (sugars, amino acids, lipids, and peptides), heavy metal ions and conjugates, xenobiotics, as well as in other biochemical and physiological processes [1,2]. The structure of these transporters is highly conserved among most eukaryotic organisms, including insects. A functional ABC transporter comprises two cytosolic nucleotide-binding domains (NBDs) that bind and hydrolyze ATP, and two hydrophobic transmembrane domains (TMDs) [3]. The NBD contains several highly conserved nucleotide-binding sequences, whereas the ABC exporter fold forms a prominent quaternary structure containing 12 transmembrane helices. In insects, the different ABC transporters have been organized into eight subfamilies (ABCA–ABCH), according to the similarity among their sequences and the organization of conserved ATP-binding cassette domains [1].

It is well-known that ABC transporters play an important role in multidrug resistance (MDR) in both bacteria and vertebrates [4]. However, knowledge on the role and function of these proteins in insects is still limited. More recently, ABC transporters have raised special interest because some genes encoding for ABC transporters, mostly of subfamilies B and C, have been linked with chemical insecticide resistance [1]. In addition, some ABC transporters were shown to serve as functional receptors for different *Bacillus thuringiensis* (Bt) insecticidal proteins [5]. Recently, the role of an ABC transporter as an importer of a digestion modulating factor was described in mosquitoes [6]. The ABC transporters were first related with Bt proteins by a genetic linkage between a mutation present in the ABC transporter subfamily C2 (*ABCC2*) of a Cry1Ac-resistant strain of *Heliothis virescens* [7]. Two years later, the functionality of the ortholog transporter as a receptor for Cry1A proteins in another insect species, *Bombyx mori*, was also confirmed [8]. Since then, a series of studies related different ABC transporters with the toxicity of Bt proteins [5].

Interestingly, ABCC3, a protein from the same subfamily as ABCC2, was also reported to play a role in determining susceptibility to Cry1 proteins in larvae from *Spodoptera exigua* [9,10], *Spodoptera frugiperda* [11], *Plutella xylostella* [12], and *Helicoverpa armigera* [13,14]. These findings suggest that ABCC2 and ABCC3 transporters, at least in some lepidopteran insects, play a role in the mode of action of Cry1 proteins from Bt. To date, research on the role of ABC transporters in *H. virescens* has exclusively focused on the ABCC2 transporter and its association with Cry1 proteins [15]. Here, we have explored the presence of paralogs to the *ABCC2* gene in *H. virescens*, identifying two new ABC transporter genes: *HvABCC3* and *HvABCC4*. In silico analysis of these proteins reveals a high similarity to the *H. virescens* ABCC2 transporter. We have further studied their possible interaction with Cry1Ac by docking analysis. The results point out that the interactions between the newly described ABCC transporters and Cry1Ac are possible, thus suggesting that they could act as receptors for Cry1A proteins.

## 2. Materials and Methods

### 2.1. Identification and Characterization of New ABCC Genes in H. virescens

To search for *ABCC2* paralogs in *H. virescens*, *ABCC* genes from a variety of phylogenetically close lepidopterans were used as templates against transcriptomic studies (from the following SRA studies: ERP021656, SRR2912076, ERP009356, SRP062666, SRP005629, SRP032396) and the genome of *H. virescens* (NWSH00000000.1, from Fritz et al. [16]). Two in silico sequences of new putative ABCC transporters were found and further characterized, which aligned with the ABCC3 and the MDR protein 4 (MDRP4) of *Helicoverpa armigera* (MW592373 and LOC110380708, respectively).

To verify the sequences, total RNA from *H. virescens* third instar larvae midguts was isolated using RNAzol reagent (MRC Inc., Cincinnati, OH, USA) according to the manufacturer’s protocol. Total RNA (2 µg) was reverse transcribed to cDNA using random hexamers and oligo (dT) following the instructions provided in the Prime Script RT Reagent Kit (Perfect Real Time from TaKaRa Bio Inc., Otsu Shiga, Japan). Then, amplification of the fragments of the *HvABCC3* and *HvABCC4* genes was performed, using 5 and 6 pairs of primers for each gene, respectively, which were previously designed with Geneious software v10 (Supplementary Table S1), according to sequences from the assemblies. Next, the sequences obtained by Sanger dideoxy sequencing were aligned to obtain the complete *HvABCC3* and *HvABCC4* genes using the same software.

### 2.2. Phylogenetic Analysis of the ABCC Transporters

The amino acid sequences of HvABCC2, HvABCC3, and HvABCC4 of 32 different transporters available from lepidopterans were aligned using MAFFT software v7 (https://mafft.cbrc.jp/alignment/software/). We have included sequences of the MDR4 proteins in the ABCC4 clade following the classification performed by Endo et al. [9]. A phylogenetic tree was generated using the neighbor-joining method in MEGA X. The percentage of replicate trees in which the associated taxa clustered together in the bootstrap test (1000 replicates) is shown next to the branches. The tree is drawn to scale, with branch lengths in the same units as those of the evolutionary distances used to infer the phylogenetic tree. The evolutionary distances were computed using the JTT matrix-based method and are in the units of the number of amino acid substitutions per site. The rate variation among sites was modeled with a gamma distribution (shape parameter = 4).

### 2.3. RT-qPCR

Relative expression levels for *HvABCC2*, *HvABCC3*, and *HvABCC4* were determined by reverse transcription quantitative polymerase-chain reaction (RT-qPCR) in a StepOnePlus real-time PCR system (Applied Biosystems, Foster City, CA, USA). Reactions were performed using 5× HOT FIREpol EVAGreen qPCR Mix Plus (ROX) from Solis BioDyne (Tartu, Estonia) and cDNA in a total reaction volume of 25 µL. Specific primers for the three ABC transporter genes and the *Rps18* gene (used as endogenous control) are shown in Supplementary Table S1. The REST MCS software v1 was used for gene expression analysis through the 2^−ΔΔCt^ method.

### 2.4. Prediction of ABC-Specific Domains and Structural Modeling

The prediction of transmembrane domains, as well as the outer and inner parts (extracellular loops and intracellular regions) of the three ABCC proteins, was performed using the DeepTMHMM server v1.0.24 (https://dtu.biolib.com/DeepTMHMM). The three-dimensional structure of the transporters was then predicted using Phyre2 software v2.0 (http://www.sbg.bio.ic.ac.uk/phyre2/html/page.cgi?id=index).

### 2.5. Docking Studies

For protein docking, the Cry1Ac monomer (ID 4ARX) and the Vip3Aa tetramer protoxin (ID 6TFJ) pdb files were downloaded from the RCSB Protein DataBank (https://www.rcsb.org). The pdb files from the three ABC transporters obtained by Phyre2 were analyzed by a Ramachandran’s plot built with the PROCHECK program, SAVES software version 6.0 (https://saves.mbi.ucla.edu) to check their suitability for docking experiments. Then, protein–protein docking simulations were performed with ClusPro 2.0 software (https://cluspro.bu.edu/login.php). In order to better define the interactions between Cry1Ac and the three ABCC transporters, the chain residues of the extracellular loops (ECLs) of the transporters and domains II and III of Cry1Ac were designated as attractant regions, since these ECLs have been previously described as the main region interacting with Bt proteins [8,17,18,19]. For Cry1A proteins, there is extensive evidence supporting the interaction of domains II and III with midgut receptors [20,21,22]. ClusPro software provides several clusters of low energy structure. The lowest scores are considered as significant interactions with less atomic contact energy [23]. The weighted scores were calculated accordingly. Results of the binding models were visualized and analyzed using USCF Chimera X v. 1.6.1 Software (https://www.cgl.ucsf.edu/chimerax/index.html).

## 3. Results and Discussion

### 3.1. Identification and Phylogenetic Analysis of Two New ABCC Transporters from H. virescens

**Figure 1:**
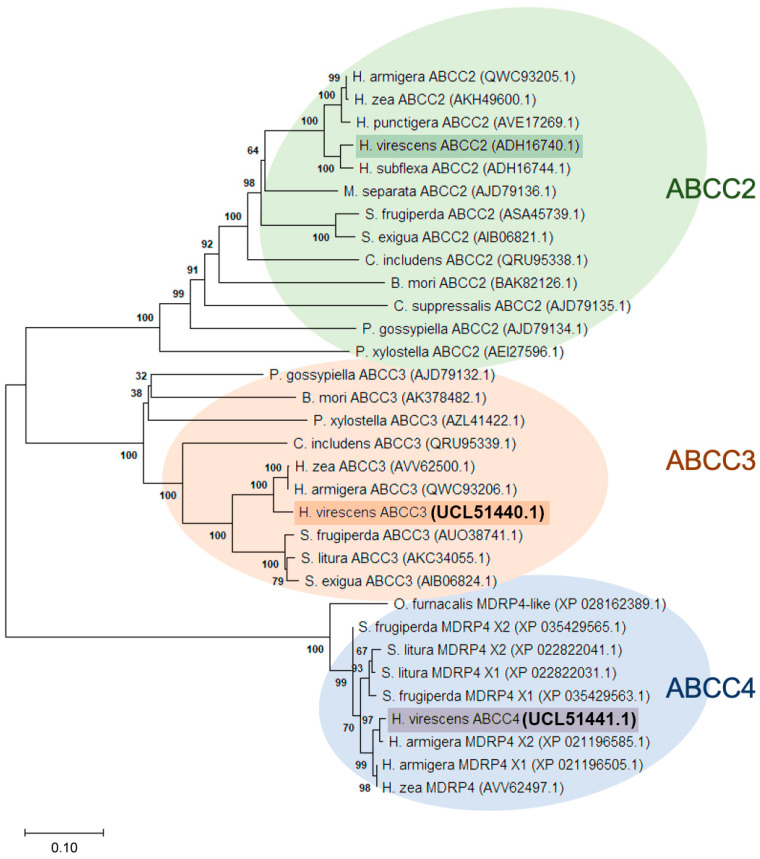
Phylogenetic tree of ABCC transporters of lepidopterans. Entire amino acid sequences of HvABCC2, HvABCC3, and HvABCC4 from lepidopterans are used for the alignment. Genbank accession numbers are shown in parentheses. Colors represent the different clades. MDRP—multi-drug resistant protein.

Previous studies have shown that ABCC2 and ABCC3 transporters from lepidopteran species act as functional receptors of some Cry1 proteins, playing a redundant role in some of them [11,12,14,24]. However, in the case of *H. virescens*, only the ABCC2 transporter has been characterized [7]. Therefore, we examined the *H. virescens* genome and data from transcriptomic studies to explore the presence of *ABCC2* paralogs. As a result, we identified two new ABC transporters that showed high similarity with other ABC transporters from phylogenetically close species of heliothines (*Helicoverpa armigera*, *Helicoverpa zea*, *Helicoverpa punctigera*, and *Heliothis subflexa*). They were named HvABCC3 and HvABCC4 because of their amino acid sequence similarity to the corresponding ABCC3 and ABCC4 clades (Figure 1). The sequences of both transporters were deposited in GenBank (UCL51440.1 and UCL51441.1, respectively). According to the analysis, the ABCC3 clade is phylogenetically closer to the ABCC2 clade than to the ABCC4 clade, since they formed different lineages from a common branch, in contrast to the ABCC4 clade. 

As seen in Figure 1, the ABCC2 clade gathers the best known members of the subfamily C transporters. This ABC transporter was first characterized because of its association with *H. virescens* resistance to Cry1A insecticidal proteins from *B. thuringiensis* [7]. Later, its role as a receptor for these proteins was demonstrated in several other lepidopterans, such as *Bombyx mori* [25], *Spodoptera litura* [26], *Spodoptera exigua* [10,27], *H. armigera* [13,14], and S. frugiperda [11]. Although ABCC2 has been widely studied over the past years, little is known about other members of the ABCC family, such as ABCC3. The relationship of the latter with Cry1A proteins was studied in different insects, such as *S. exigua*, *S. litura*, *H. armigera,* and *P. xylostella* [9,10,11,12,13,14]. In *H. virescens*, we hypothesize that the HvABCC3 described here also might play a redundant role in acting as a Cry1-protein receptor due to its high similarity with that of *H. armigera*.

In regard to the ABCC4 clade, no other ABCC4 ortholog has been characterized in the Noctuidae family besides the HvABCC4 described in this study, probably because it has gone unnoticed as another member, due to its high sequence similarities, or because it was described only as an MDR protein. Thus, according to the classification by Endo et al. [10], other new ABCC4 members might arise for *Ostrinia furnacalis*, *S. frugiperda*, *S. litura*, *H. armigera,* or *H. zea*. 

### 3.2. ABCC Transporters Are Differentially Expressed in the Midgut

**Figure 2:**
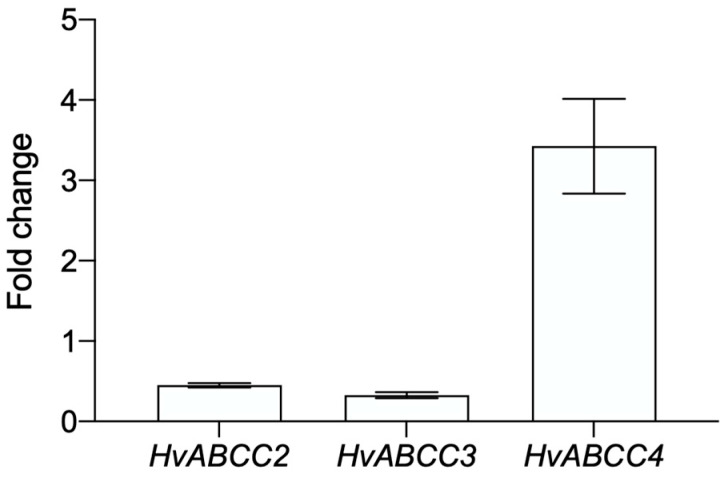
Expression profile analysis of the three ABC transporters in midguts of *H. virescens* 3rd instar larvae. The transcript levels of the *Rps18* gene were used as a reference. Bars represent the mean of three independent biological replicates (±SD).

The expression levels of *HvABCC2*, *HvABCC3,* and *HvABCC4* genes were analyzed in the midgut from 3rd instar larvae by RT-qPCR. The *Rps18* gene (ribosomal protein S18) was used as endogenous reference control. For *HvABCC2* and *HvABCC3*, both showed similar expression levels, while *HvABCC4* levels were expressed 8-fold higher (Figure 2). 

To our knowledge, few studies have compared the basal expression levels of different members of the ABCC family in the midgut of lepidopteran larvae. In several Lepidoptera, similar expression levels between the *ABCC2* and *ABCC3* transporter genes have been found, mainly in midguts of *S. exigua*, *B. mori*, *S. frugiperda*, and *Mythimna separata* [9,11,28,29], and whole larvae of *Chrysodeixis includens* [30]. Our results are in agreement with previous studies due to the similarity of expression levels between *HvABCC2* and *HvABCC3*, while the higher expression of *HvABCC4* could point out a possible relevance of the latter in midgut cell detoxification.

### 3.3. In Silico Analysis of the ABCC Proteins Reveals High Similarities on Their Structures

**Figure 3:**
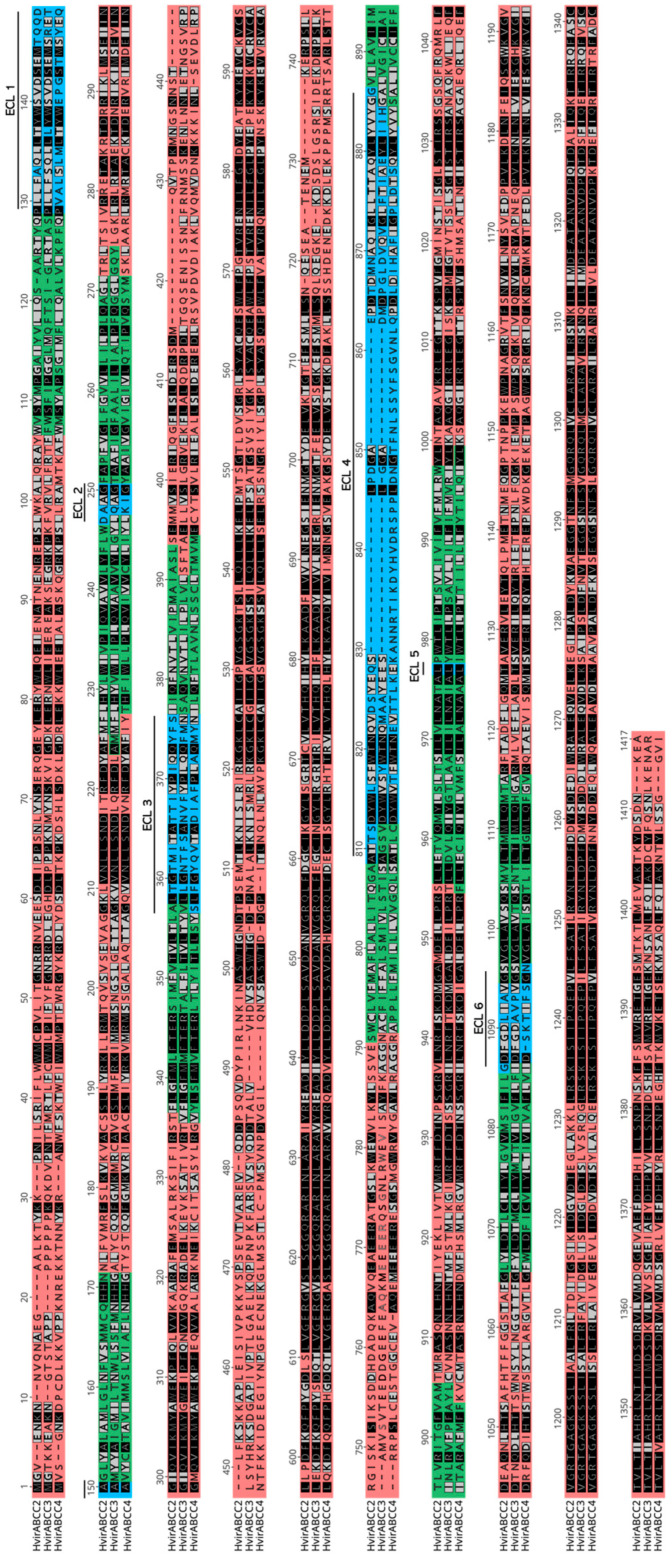
Alignment of the amino acid sequences and predicted topology of the three *H. virescens* ABCC transporters. Multiple alignments of HvABCC2 (ADH16740.1), HvABCC3 (UCL51440.1), and HvABCC4 (UCL51441.1) were performed with MAFFT software. Identical and similar residues are highlighted in black and grey, respectively. Topology was predicted by DeepTMHMM 1.0.24 software: Intracellular regions are shown in red, transmembrane helices in green, and extracellular loops (ECLs) in blue.

The protein topology (Figure 3) and the three-dimensional structure of the new ABCC transporters was characterized and compared to that of HvABCC2. Although the amino acidic length is slightly different (1339, 1369, and 1394 amino acids for HvABCC2, HvABCC3, and HvABCC4, respectively), their topology is highly similar.

In our study, the prediction of these transmembrane helices showed two differentiated TMDs, each of them with three regions facing the outer part of the membrane that correspond to the ECLs (Figure 3) found in other ABCC transporters. Interestingly, the position and the length of each one of the six ECLs is relatively well conserved among the three transporters, suggesting a possible functional redundancy on their biological role, with the exception of ECL4 in HvABCC4, where the greatest differences compared to the HvABCC2 and HvABCC3 are observed. It is worth to note that these ECLs are involved in blocking the access of allocrites through the channel in the closed state [31]. Interestingly, it was recently described how the *H. virescens* ABCC4 characterized in this study shows homology to an ABC transporter previously described in mosquitoes [32]. This ABC transporter, known as AeaTMOF receptor, acts as an importer of a hormone involved in digestion control in the midgut of mosquitoes [6], and it was suggested to play a similar role in *H. virescens* [32]. Therefore, it is worthwhile to elucidate the role of ABC transporters in insects, as they may have a dual role in the response mechanisms to Bt, either acting as putative receptors or being involved in the processing response of Bt proteins during gut digestion. These steps in the response (midgut activation of Bt proteins by digestion, and binding to receptors) are of paramount importance in the use of Bt as a bioinsecticidal agent [33].

Besides this biological function, the ECLs are the regions of the transporters that interact with Bt proteins [5]. It was reported that a single amino acid mutation in the second ECL from the ABCC2 of *B. mori* conferred resistance to the Cry1Ab protein [8] and that insertions in this ECL could disrupt its Cry1Ab or Cry1Ac receptor function [18]. Later, it was shown that the first and fourth ECLs of the same transporter were involved in the action of Cry1Aa [19]. Similarly, it was observed that a single amino acid in the first ECL of the ABCC2 of *S. frugiperda* and *H. armigera* could mediate Cry1Ac toxicity [17]. In the case of the ABCC3, which has been less explored, the substitution in ECL1 with amino acids from the ABCC2 conferred cell susceptibility to Cry1Ab and increased susceptibility to Cry1Aa [28]. For these reasons, the sequence, length, and similarity of amino acids present in these ECLs have shown to be relevant in the interaction with Bt proteins. Therefore, the interactions between the specific sequences of each ECL and Bt proteins are still needed to be characterized.

### 3.4. Prediction of Possible Interactions between Transporters and Bt Proteins

**Figure 4:**
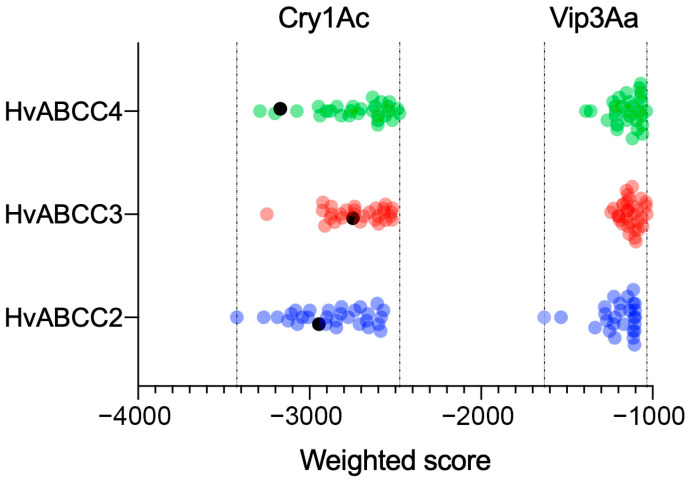
Weighted score values obtained by docking of the three ABC transporters with Cry1Ac proteins. The first 30 clusters for each transporter–protein interaction are represented, and each dot represents the weighted score for a given cluster. The black dots are the weighted scores of the clusters selected for the schematic representation in Figure 5. Weighted scores represent the center energy of the balanced model obtained by ClusPro 2.0 software.

In *H. virescens*, it is well-known that the ABCC2 transporter acts as functional receptor to Cry1A proteins (Cry1Aa, Cry1Ab, and Cry1Ac) [15]. Here, in order to determine whether the two newly described ABCC members can also interact with Cry1Ac, docking analysis was performed. As a proof of concept of this methodology, the interaction between the *H. virescens* ABCC2 transporter and Cry1Ac was analyzed. The ClusPro program generated all possible binding models for the three *H. virescens* ABC transporters and Cry1Ac, and then clustered the most favorable binding models. The weighted scores for the interaction of Cry1Ac and the three transporters are shown in Figure 4. Similarly weighted score values were obtained for the three ABCC transporters and Cry1Ac, though the ABCC2 transporter had the lowest weighted scores, indicating that the interaction is most favorable. Although it is widely accepted that ABCC2 and ABCC3 transporters are functional receptors of Cry1 proteins in lepidopterans, the contribution of ABCC2 in determining larval Cry1A susceptibility has been shown to be higher than that of ABCC3 [10,11]. Accordingly, the lowest weighted scores found in this study for HvABCC2 compared to HvABCC3 might indicate that the contribution of this receptor in the mode of action of Cry1Ac in *H. virescens* is more relevant than the other two ABCC receptors.

To reinforce the docking results, we analyzed the possible interaction of the three *H. virescens* ABCC transporters and Vip3Aa, a toxic Bt protein known not to share binding sites with Cry1 proteins [34,35]. The weighted scores for the interaction of Vip3Aa and the three transporters were much higher (Figure 4), compared with those of Cry1Ac, as one would expect for a protein not binding to these membrane proteins. These data support the docking predictions in that Cry1Ac very likely could bind to HvABCC3 and HvABCC4.

**Figure 5:**
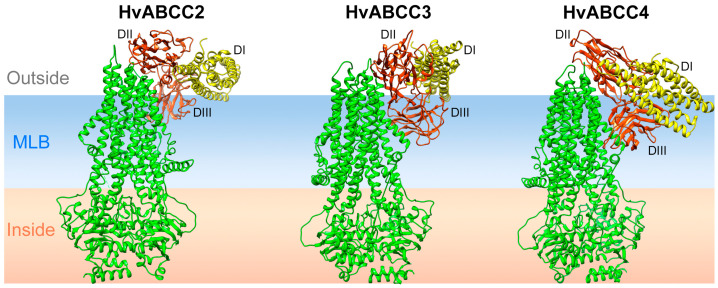
Schematic representation of the Cry1Ac protein docked to the three ABCC transporters. One cluster model for each transporter–protein interaction was chosen for the representation (black dots in Figure 4). MLB—membrane lipid bilayer; green—ABCC transporters; yellow—domain I of Cry1Ac; red—domains II–III of Cry1Ac.

The activated Cry1A proteins are formed by three domains [21]. Domain I is reported to function in membrane insertion [36], whereas domains II and III are implicated in the receptor binding [37,38]. Figure 5 shows a model selected as representative of the interaction between the Cry1Ac protein and the *H. virescens* ABCC2, ABCC3, and ABCC4 transporters. Our docking models predict that the ECLs of the ABCC transporters may bind the Cry1Ac protein through domain II, in agreement with experimental results from previous studies in *B. mori* and *S. exigua* [18,27].

## 4. Conclusions

In a search for *HvABCC2* paralogs in *H. virescens*, the analysis revealed two genes which were named *HvABCC3* and *HvABCC4* based on their amino acid sequence similarity to the corresponding ABCC3 and ABCC4 clades. The protein topologies of the three *H. virescens* ABC transporters were found to be highly similar. As in HvABCC2, the new ABC transporters contain two cytosolic ATP-binding domains and two transmembrane domains, each of them with six membrane-spanning helices and three extracellular loops.

Considering the well-known role of the HvABCC2 transporter as a functional receptor for Cry1A proteins, the docking results suggest that the new HvABCC3 transporter could also have a redundant role as the receptor for these proteins, as observed in other insect species. Functional studies of HvABCC3 and HvABCC4 should finally unravel whether these putative transporters are further involved in the mode of action of insecticidal Bt proteins.

## Data Availability

Data are contained within the article and Supplementary Materials.

## Supplementary Materials

The following supporting information can be downloaded at: https://www.mdpi.com/article/10.3390/biom14040397/s1, Table S1: Primers used in PCR and sequencing of *HvABCC3* and *HvABCC4*, and qPCR of *Rps18*, *HvABCC2*, *HvABCC3* and *HvABCC4* from *Heliothis virescens.*
