# Supplementary material for: New Paralogs of the Heliothis virescens ABCC2 Transporter as Potential Receptors for Bt Cry1A Proteins
Source: Biomolecules. 2024 Mar 26;14(4):397. doi: 10.3390/biom14040397 (PMC11047971; doi:10.3390/biom14040397)
Supplement: Supplementary file 1 [file biomolecules-14-00397-s001.zip › biomolecules-2940709-supplementary.pdf]

Supplementary Table S1. Primers used in PCR and sequencing of *HvABCC3* and *HvABCC4*, and qPCR of *Rps18*, *HvABCC2*, *HvABCC3* and *HvABCC4* from *Heliothis virescens*.

Function	Target	Name	Sequence (5'-3')
Sequencing	***HvABCC3*** **gene**	HvABCC3_5UTR3_F	GCGGAACTCTCCACATTTGC
		HvABCC3_526_R	CATCTTCATACCGAACTGCTGGC
		HvABCC3_356_F	GGCATCTCCATTGCTGTTTAGTC
		HvABCC3_1440_R	CAAAGCAGTGTCGTCCTGGTATG
		HvABCC3_1331_F	ATCGCAAATCGGATGGAGC
		HvABCC3_2272_R	GGTTGCCTGACTGCCTCTCC
		HvABCC3_2153_F	GAAGCATTGATGAGAAGGACAGGC
		HvABCC3_3468_R	TATCAGGGAAGATTTGCCAGC
		HvABCC3_3377_F	AGCCCGTATTGAAGAACCTGAAC
		HvABCC3_3UTR3_R	TGCCTACTCTACATTACTATTCACAAG
Sequencing	***HvABCC4*** **gene**	HvABCC4_5UTR2_F	GGACTCTAAGCCCGAGGACAT
		HvABCC4_552_R	GAGTGAGCAGGCGGCGATG
		HvABCC4_464_F	TGTCGCTGGTCATCGCCTTC
		HvABCC4_1381_R	TGCACTCGAATCCAGGGTTG
		HvABCC4_1219_F	CGAGAGGATCTCCGCAGTGTG
		HvABCC4_2080_R	CCCTGTGCTCACTAATTCGTCG
		HvABCC4_1970_F	TCCTCGTCACGCATCAACTC
		HvABCC4_2794_R	ATTCATCGAGGGCTCCAATGTC
		HvABCC4_2673_F	CCGCAACCTCCACAACGAC
		HvABCC4_3611_R	CAACGTCGTCAATGAGCACTTC
		HvABCC4_3448_F	ACGCCCGAAGACTTGCCAG
		HvABCC4_3UTR5_R	ACGAAACTACATAGAGAAGGACACTG
qPCR	***Rps18*** **gene**	Rps18_Forw	ATGGCAAACGCAAGGTTATGTTT
		Rps18_Rev	TTGTCAAGATCAATGTCGGCTTT
	HvABCC2 **gene**	HvABCC2_Forw	TGCGCTAGCCATGCTGGGACT
		HvABCC2_Rev	ACCGACACTTGAGTCATGCGGAGCA
	HvABCC3 **gene**	HvABCC3_Forw	CCACGGTGCACTGTATTGCC
		HvABCC3_Rev	CCGTCGTCTCACCCAGTGAA
	HvABCC4 **gene**	HvABCC4_Forw	GGCGAGCAAGCAGGGCCGTA
		HvABCC4_Rev	GCTAACGCCACTGGTTGGAAAGGC
